# Supplementary material for: The Synergistic Effect of GH13 and GH57 GBEs of Petrotoga mobilis Results in α-Glucan Molecules with a Higher Branch Density
Source: Polymers (Basel). 2023 Dec 2;15(23):4603. doi: 10.3390/polym15234603 (PMC10708623; doi:10.3390/polym15234603)
Supplement: Supplementary file 1 [file polymers-15-04603-s001.zip › polymers-2634722-supplementary.pdf]

Supplementary data

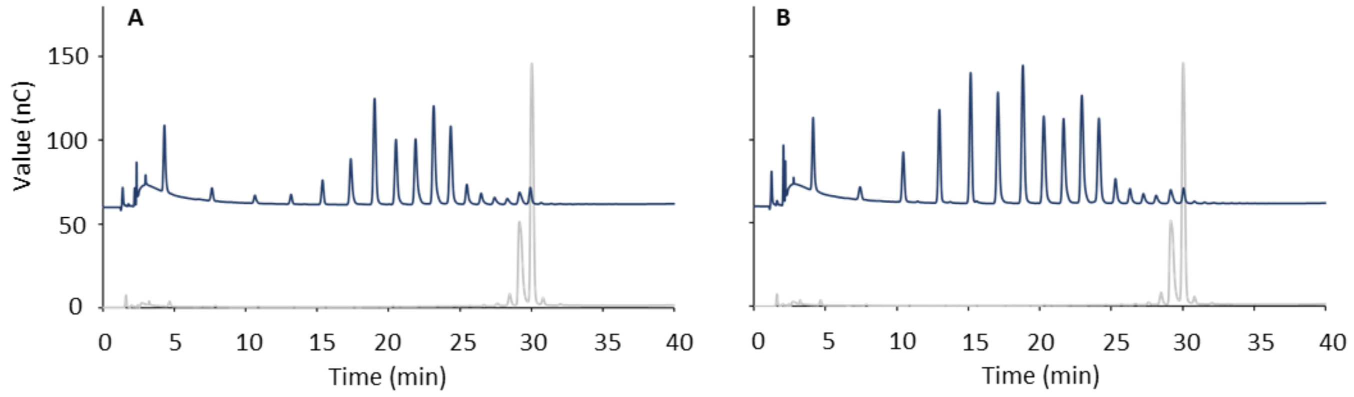

Figure S1. Chain length distribution of MD18 modified with PmGBE13 (*Petrotoga mobilis;* 1U^B/g S) for 24 hours before [A] and after [B] debranching (blue) compared to the untreated substrate (grey). Y axis are the same hight and spacing nC intensity value for A and B.

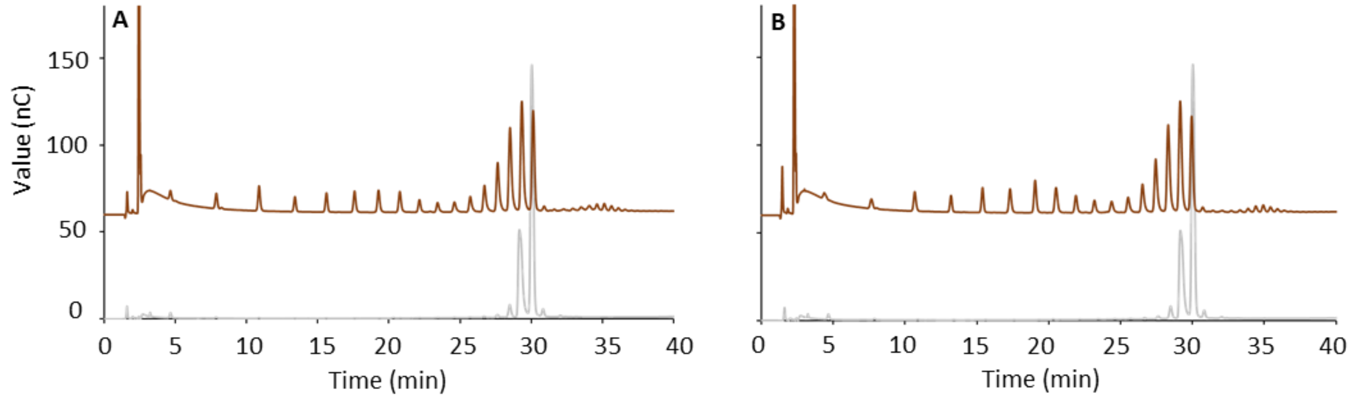

Figure S2. Chain length distribution of MD18 modified with PmGBE57 (*Petrotoga mobilis;* 1U^B/g S) for 24 hours before [A] and after [B] debranching (orange) compared to the untreated substrate (grey). Y axis are the same hight and spacing nC intensity value for A and B.

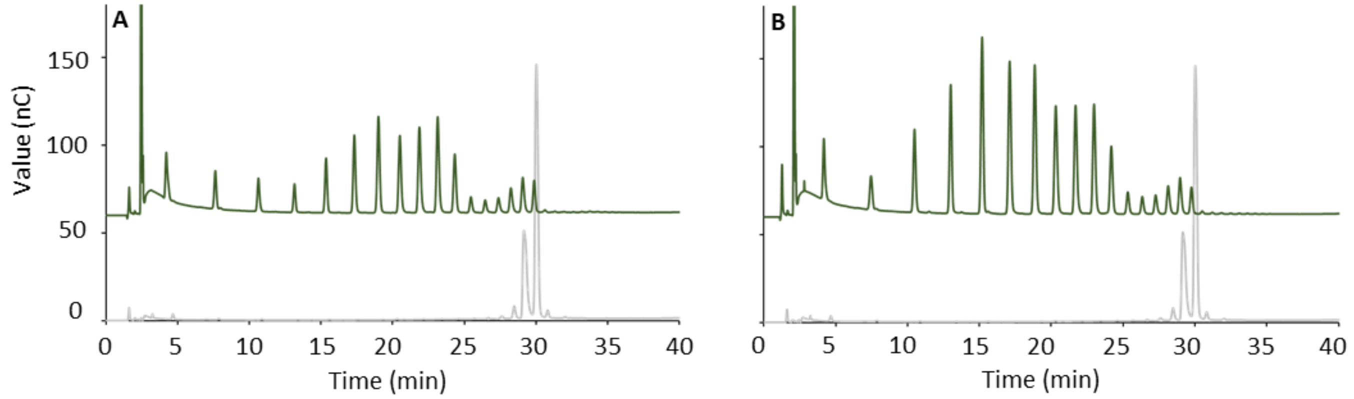

Figure S3. Chain length distribution of MD18 modified with PmGBE13 and PmGBE57 (*Petrotoga mobilis;* 1U^B/g S) in a one-step modification for 24 hours before [A] and after [B] debranching (green) compared to the untreated substrate (grey). Y axis are the same hight and spacing nC intensity value for A and B.

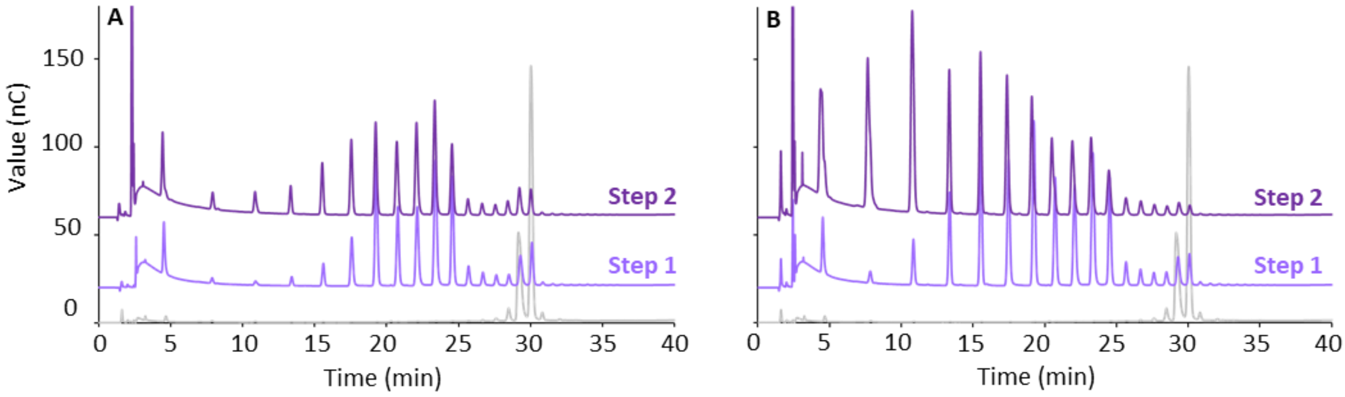

Figure S4. Chain length distribution of MD18 modified with PmGBE13 (step 1) and PmGBE57 (step 2) (*Petrotoga mobilis;* 1U^B/g S) in a two-step modification for 24 hours before [A] and after [B] debranching (purple) compared to the untreated substrate (grey). Y axis are the same hight and spacing nC intensity value for A and B.

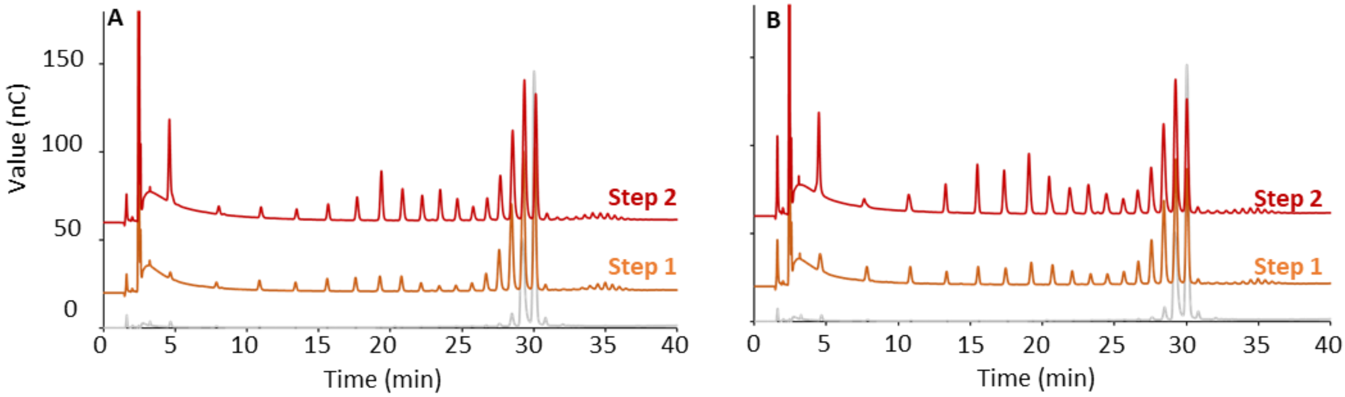

Figure S5. Chain length distribution of MD18 modified with PmGBE57 (step 1) and PmGBE13 (step 2) (*Petrotoga mobilis;* 1U^B/g S) in a two-step modification for 24 hours before [A] and after [B] debranching (orange/red) compared to the untreated substrate (grey). Y axis are the same hight and spacing nC intensity value for A and B.
